# Effect of sleep ambient music on sleep quality and mental health in college students: a self-controlled study

**DOI:** 10.3389/fpsyg.2023.1171939

**Published:** 2023-07-07

**Authors:** Shun-Ping Hu, Ya-Meng Yang, Wen-Hao Chen, Shan-Shan Lu, Tong Niu, Yun-Zhu Xia, Jin-Yi Li

**Affiliations:** ^1^Department of Medical Humanities, School of Basic Medicine, Army Medical University, Chongqing, China; ^2^Department of Southwest Hospital, Army Medical University, Chongqing, China; ^3^School of Clinical Medicine, Army Medical University, Chongqing, China

**Keywords:** sleep ambient music, music intervention, sleep quality, metal health, anxiety, depression, college students

## Abstract

To verify the effect of sleep ambient music intervention (SAMI) on sleep quality and mental status of college students, and to further explore the minimum effective duration of SAMI, this study was designed as a pre-and post-intervention self-controlled exploratory study. Participants were subjected to a one-week no-intervention test, then 4 weeks of music intervention followed. Subjective sleep quality data were collected using the Pittsburgh Sleep Quality Index (PSQI); objective sleep quality data were collected with Actigraphy; and mental status data were collected using the State–Trait Anxiety Inventory (STAI) and the Beck Depression Inventory-II (BDI-II). Data were analyzed and processed using mixed-effects models and repeated measures. The results showed that compared with the no-intervention week, college students’ subjective sleep quality, objective sleep onset latency (SOL), trait anxiety, and depression symptom were reduced at week 1; week 2; week 3; week 4 under SAMI; state anxiety of college students at week 3 and week 4 under SAMI were also reduced. And there were differences in sleep quality among college students of different genders too. Compared with females, males had worse sleep efficiency (SE), shorter total sleep time (TST), and more awaking times (AT). In addition, 3 days was the minimum effective length for SAMI to shorten objective SOL, and 2 days was the minimum effective length to shorten the subjective SOL of college students. The findings of this study suggest that SAMI can improve subjective sleep quality, shorten objective SOL, and reduce anxiety and depression in college students. Interventions for more than 3 days had a significant effect on shortening SOL and long-term effects seemed to emerge after 3 weeks.

## Introduction

Sleep is an important criterion reflecting the physical, mental, and social health of a person. The university stage is important in the development of individuals in their young adulthood, a critical period for gradual physical and mental maturation and the improvement of learning ability and general competence (Feldman, 2017). Good sleep can regulate college students’ emotions (Scott et al., 2021), consolidate memory (Trouche et al., 2020), improve their learning ability, and promote their physical and mental health. While lack of sleep will not only increase the risks of obesity (Anam et al., 2022) and cardiovascular and cerebrovascular diseases (Li et al., 2022), it will also trigger anxiety, depression, and other mental disorders (Ward et al., 2014) and even lead to a decrease in cognitive levels, such as attention (Muto et al., 2016; Di Muzio et al., 2020) and memory (Ward et al., 2014). Sleep problems have now become one of the main factors affecting the physical and mental health of college students (Freeman et al., 2017), mainly manifested by difficulty in falling asleep (Reis et al., 2021), poor sleep quality (Chowdhury et al., 2020), and disrupted sleep patterns (Li T. et al., 2020). Several national surveys have shown that sleep problems are more prominent among college students. The percentage of college students reporting disturbed sleep patterns was 86% (Lund et al., 2010). More than 60% of college students had poor sleep quality (Lund et al., 2010) and 30% were clinically evaluated for insomnia (Becker et al., 2018). In addition, sleep problems can affect the mental health and cognitive level of college students (Mason et al., 2021). They can even lead to psychiatric disorders. Studies have shown that the sleep problems of college students not only increase the risk of anxiety and depressive symptoms (Ramos et al., 2021) but also lead to lower grades and graduation rates among college students (Chen and Chen, 2019) and decreased well-being (Fischer et al., 2020). In the past 3 years, due to the impact of COVID-19 on life, study, and employment, negative emotions (Xiang et al., 2020) and stress (Beiter et al., 2015) were prevalent, making sleep problems more widespread among college students (Xu et al., 2022; Zhang et al., 2022). Some studies on college students have found that gender and grade also have an impact on their sleep quality (Ghrouz et al., 2019; Sundas et al., 2020).

Currently, there are two main types of treatment for sleep problems: pharmacological and non-pharmacological. Pharmacological interventions can quickly solve some sleep problems, while the disadvantage is that they can lead to dependence, and long-term medication carries the risk of triggering cognitive impairment (Lee et al., 2020). Common non-pharmacological approaches include cognitive behavioral therapy for insomnia (Carney et al., 2017), positive meditation (Rusch et al., 2019), and transcranial magnetic stimulation (Nardone et al., 2020), which can improve sleep quality. However, these approaches can have long intervention cycles, are highly dependent on professional guidance, equipment (Miao et al., 2020; Wang et al., 2021), and on subject compliance. As a special group, college students need a simpler and more effective non-pharmacological intervention to urgently resolve their increasing sleep problems (Acker and Cordi, 2019).

Music is a very popular way to have fun and relax in public, especially among college students (Trahan et al., 2018). As a non-pharmacological intervention, listening to music (Jespersen et al., 2022) has been recommended as one of the sleep quality promotion modalities by the American Sleep Association (Gradisar et al., 2013). Several studies have shown a significant effect of music on improving sleep quality (Yamasato et al., 2020), increasing sleep satisfaction (Dos, 2022), and reducing depression (Harmat et al., 2008) in college students. However, some studies reported that music could not affect objective sleep quality (Lazic and Ogilvie, 2007; Messineo et al., 2017). In summary, most of the positive results about music interventions for sleep quality were subjective, but the objective evidence is controversial. Previous studies had used classical (Burrai et al., 2020), light (Hu et al., 2015), ethnic (Cai et al., 2015), and religious music (Huang et al., 2017) to improve sleep quality, all with certain efficacy, but because of the inconsistent populations, the wide range of music styles, and the varied basis for music selection, either by the researcher or by the subject, resulting in a high degree of individualization of the results, and no unified standard could be formed yet (Iwaki et al., 2003; Wang et al., 2016). The length of the interventions ranged from 1 day to 4 weeks (Yamasato et al., 2020), resulting in an unguaranteed validity of the results. The exploration of the minimum effective intervention duration determines whether a standardized treatment regimen can be formed.

In 2018, Trahan et al. used some new genres of music, such as ambient and meditation music, in a large sample cross-sectional survey on music and sleep (Trahan et al., 2018). In a large-sample music analysis study by Scarratt (2021), ambient music including white noise and natural ambient sounds were also considered as representative genres in sleep music (Scarratt, 2021). Current clinical studies on sleep interventions with ambient music are scarce, and the effects are not yet clear. By analyzing the sleep music used in previous studies, we found similar characteristics and filtered out a type of music that can describe a specific environment with particular musical elements, namely “sleep ambient music.” In this study, we defined sleep ambient music as a form of music that describes a particular ambiance with particular musical elements. Listening to this style of music can lead people to imagine an aspect of nature or a specific environment, create relaxation, and then succeed in promoting sleep. The music contains instrumental and natural (such as rain and waves) or environmental (such as coffee house and city street) sound elements. Compared to other music, sleep ambient music has stricter criteria in terms of musical parameters (low loudness, weak energy, slow tempo, flat melody, etc.), which makes it more consistent and likely to be a more standardized music choice, which will help improve the reproducibility of the results. Therefore, to address the shortcomings in the field of music intervention for sleep quality (less objective evidence, lack of standardization in music selection, and unclear effective intervention duration), this study collected objective sleep quality data from college students by Actigraphy and used sleep ambient music as an intervention to investigate the effects of sleep ambient music intervention (SAMI) on sleep quality and mental health of college students, as well as to explore the minimum effective intervention duration of SAMI to provide systematic theoretical support.

## Materials and methods

### Study population

College students with poor sleep quality (PSQI ≥8) were recruited for the study from June 2020 to September 2021. The study was approved by the University Institutional Review Board (2019 No. 002–02). Participants signed a written informed consent form. **Inclusion criteria** (1) college students aged 18 to 24 years; (2) Pittsburgh Sleep Quality Index (PSQI) scale score ≥ 8. **Exclusion criteria** (1) hearing impairment; (2) impaired cognitive function; (3) using sleep-influencing drugs (e.g., cold medicine, melatonin, etc.) during the experiment; (4) drinking alcohol or caffeine 2 h before bedtime; (5) receiving other sleep intervention modalities (e.g., positive thinking, cognitive behavioral therapy, etc.); (6) allergic to the experimental equipment. **Withdrawal criteria** (1) non-compliance of trial protocol after inclusion; (2) incomplete data, affecting the judgment of efficacy. (3) voluntarily withdrawing from the trial; (4) sleep problems deteriorated during the trial; (5) necessary abeyance in the judgment of the investigator.

### Power analysis

Using SOL as the primary outcome indicator and referring to Cordi’s study, the SOL of the subjects was 12.31 ± 3.12 min without intervention and 11.08 ± 2.36 min with music intervention, setting bilateral α = 0.05 and 1-β = 0.90, and using PASS 15 to obtain a sample size of N = 65 cases. Considering a 20% loss of follow-up, at least 73 cases need to be included in this study.

### Study methods

(1) General information questionnaire: Designed by the investigator which includes name, gender, age, grade, sleep problems, factors affecting sleep, and ways to cope with sleep problems. (2) Sleep log: Subjective indicators (SOL, SE, TST, AT) were collected by the National Sleep Foundation Sleep Diary (NSFSD), which includes last night’s bedtime, last night’s sleep time, wake time, number, and time of nighttime body movements, etc. (3) ActiGraph (model: wGT3X-BT, USA), Measuring objective indicators (SOL, SE, TST, AT). (4) Pittsburgh Sleep Quality Index, PSQI (Veqar and Hussain, 2017): 18 scoring entries were used to assess the sleep quality of the subjects in the last month. Based on the entries, sleep quality was divided into seven factors: sleep quality, sleep latency, sleep persistence, sleep efficiency, sleep disorder, medication, and daytime dysfunction. Each factor was scored on a scale of 0–3. The PSQI score ranged from 0 to 21, with a total score of ≥8 indicating poor sleep quality and < 8 indicating acceptable one. (5) State–Trait Anxiety Inventory, STAI: using the version prepared by Charles D. Spielberger et al., and translated by the Chinese scholar Zheng Xiaohua et al. There are 40 items, and each item is rated on a scale of 1 to 4. Items 1 to 20 are the State Anxiety Inventory (S-AI), which corresponds to current feelings. Items 21 to 40 are the Trait Anxiety Inventory (T-AI), which corresponds to usual feelings. The cumulative value of state or trait anxiety ranges from 20 to 80, with higher scores reflecting higher levels of state or trait anxiety. (6) Beck Depression Inventory-II, BDI-II (Wang and Gorenstein, 2013). This is used to measure the degree of depression, with 21 entries, each rated on a scale of 0 to 3, with a score range of 0 to 63. A score of 0 to 13 indicates no depression, 14 to 19 indicates mild depression, 20 to 28 indicates moderate depression, and 29 to 63 indicates severe depression.

### Study program

**Figure 1:**
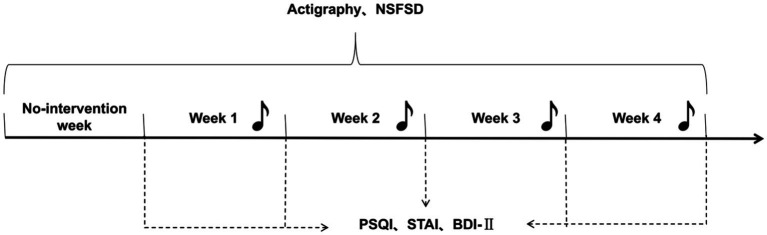
Experimental flow chart. Pittsburgh Sleep Quality Index, PSQI; State–Trait Anxiety Inventory, STAI; Beck Depression Inventory-II, BDI-II. Daily wearing of Actigraphy and completion of sleep log; weekly completion of PSQI, STAI, BDI-II.

This was pre-and post-intervention self-controlled study, and subjects were located at dormitory completion. The study was conducted using PSQI and SOL as the primary efficacy indicators and TST as the safety indicator. Actigraphies were worn throughout the whole test to collect objective indicators, and the sleep log was filled out upon waking up each day. PSQI, STAI, and BDI-II were collected on the last day of each week. In no intervention week, subjects maintained their daily routine without any intervention. During the SAMI weeks, on the first day of each week, the subject chose an acceptable music that will be distributed each night before they went to sleep. The selected music will be removed from the following week’s selection to ensure that subjects receive a different sleep ambient music intervention each week (Figure 1).

### Blind method

A single blind design was adopted in this study. (1) Data recorders, and data counters were unaware of the music distribution. (2) At the end of the intervention, none of the investigators involved in the evaluation of the effect of the music intervention and the safety evaluation was aware of the treatment factors.

### Music filtering and play methods

Based on the characteristics of sleep ambient music summarized in previous literature, the music was screened from the library of online music platforms (e.g., NetEase, CloudMusic, and Himalayan Music). Music was reviewed by two researchers with music-related professional backgrounds and two researchers with music and sleep-related knowledge, resulting in 152 pieces of eligible intervention music. All music was in mp3 format with a bit rate of 320 kbps. The intervention music played in a continuous loop through a WeChat app (online sleep platform) developed by our team, subjects used a Bluetooth headband to listen to the music, the volume level is controlled by themselves, and the music will be turned off after 30 min automatically.

### Statistical analysis

The data of the sleep intervention experiment were analyzed by linear mixed effect model, with SOL, SE, TST, AT, PSQI, STAI, BDI as response variables and Time (week), Gender, and Grade as independent variables to explore the effects of SAMI on the sleep quality and mental health status of college students. The linear mixed effect model is an extension of the general linear model (Verbeke and Molenberghs, 2001), which includes fixed effects and random effects.


γ=xβ+Ζy+ε

Using repeated measures to compare the effects of interventions at different time points. Time (day) was used as the explanatory variable; subjective SOL and objective SOL were used as response variables to explore the minimum effective time of SAMI needed to improve the sleep quality of college students. SPSS26 statistical analysis software was used, expressing counted data as percentages (%). Measurement data were expressed as $\bar{\chi }\pm s$ , and the mixed-effects models and repeated measures were tested at α = 0.05.

## Results

### Demographic information

A total of 154 college students were recruited, 9 subjects were eliminated due to non-compliance of trial protocol, and the remaining 7 dropped out. A total of 138 students were finally enrolled, with an average age of 20.029. Of the students enrolled, 52.9% were male and 47.1% were female. Freshmen accounted for 29.7%, sophomores accounted for 62.3%, and juniors accounted for 8.0%. Most of the students suffered from difficulty in falling asleep, which is the main sleep problem, accounting for 45.7%, and the top two factors affecting sleep were stress (32.6%) and psychological factors (26.2%). Most of the college students chose to listen to music (31.1%) and play with mobile phones (28.1%) as their coping measures. The average scores of PSQI, S-AI, T-AI, and BDI were 8.928, 44.333, 46.022, and 11.159, respectively (Table 1).

**Table 1:** Characteristics of respondents.

Characteristics (*n* = 138)	
Age; mean (SD)	20.029 (1.255)
*Gender*	
Male	73 (52.9%)
Female	65 (47.1%)
*Grade*	
Freshman	41 (29.7%)
Sophomore	86 (62.3%)
Junior	11 (8.0%)
*PSQI; mean (SD)*	8.928 (2.866)
*Emotion related indicators; mean (SD)*	
S-AI	44.333 (10.456)
T-AI	46.022 (9.500)
BDI-II	11.159 (9.355)

SD refers to standard deviation.

### Effect of SAMI on sleep quality

In terms of subjective sleep quality, compared with the no-intervention week, SOL, SE, TST, AT at week 1 (β = −2.031, *p* = 0.015; β = 1.103, *p* < 0.001; β = 14.624, *p* < 0.001; β = −0.101, *p* = 0.007, respectively); week 2 (β = −2.468, *p* = 0.003; β = 1.509, *p* < 0.001; β = 8.715, *p* = 0.001; β = −0.122, *p* = 0.001, respectively); week 3 (β = −3.181, *p* < 0.001; β = 1.597, *p* < 0.001; β = 10.547, *p* < 0.001; β = −0.191, *p* < 0.001, respectively); and week 4 (β = −5.146, *p* < 0.001; β = 2.407, *p* < 0.001; β = 7.862, *p* = 0.004; β = −0.225, *p* < 0.001, respectively) under SAMI were statistically different, showing that the SAMI significantly reduced SOL, AT and improved SE, TST in college students. PSQI scores were statistically different in weeks 1 to 4 (β = −0.739, *p* < 0.001; β = −1.054, *p* < 0.001; β = −1.391, *p* < 0.001; β = −1.511, *p* < 0.001, respectively) under intervention compared to the no-intervention week, meaning that SAMI significantly reduced PSQI scores among college students (Table 2).

**Table 2:** Linear mixed effect module analysis results-subjective sleep quality.

	SOL		SE		TST		AT		PSQI	
	*β*	*t* value	*β*	*t* value	*β*	*t* value	*β*	*t* value	*β*	*t* value
Constant	19.670	13.345^***^	91.648	170.366^***^	364.970	77.277^***^	0.593	8.738^***^	7.275	9.885^***^
*Time (week)*										
No-intervention	0.000	–	0.000	–	0.000	–	0.000	–	0.000	–
1	−2.031	−2.441^*^	1.103	3.625^***^	14.624	5.392^***^	−0.101	−2.709^**^	−0.739	−3.980^***^
2	−2.468	−2.967^**^	1.509	4.957^***^	8.715	3.213^**^	−0.122	−3.262^**^	−1.054	−5.677^***^
3	−3.181	−3.823^***^	1.597	5.247^***^	10.547	3.889^***^	−0.191	−5.125^***^	−1.391	−7.492^***^
4	−5.146	−6.185^***^	2.407	7.910^***^	7.862	2.899^**^	−0.225	−6.033^***^	−1.511	−8.136^***^
*Gender*										
Male	−0.545	−0.919	0.071	0.328	1.101	0.571	0.001	0.018	−0.199	−0.497
Female	0.000	–	0.000	–	0.000	–	0.000	–	0.000	–
*Grade*										
Freshman	−0.918	−0.747	0.499	1.111	−1.797	−0.450	−0.034	−0.626	0.649	0.819
Sophomore	−0.051	−0.039	0.299	0.626	−4.125	−0.976	−0.071	−1.222	1.186	1.584
Junior	0.000	–	0.000	–	0.000	–	0.000	–	0.000	–

SOL, Sleep Onset Latency; SE, Sleep efficiency TST, Total sleep time; AT, Awaking times; T, Pittsburgh Sleep Quality Index; PSQI. ^*^p<0.05; ^**^p<0.01; ^***^p<0.001.

In terms of objective sleep quality, compared to the no-intervention week, SOL at weeks 1 to 4 (β = −1.274, *p* < 0.001; β = −1.172, *p* = 0.001; β = −1.658, *p* < 0.001; β = −1.624, *p* < 0.001, respectively) under SAMI and SE at interventional week 3 (β = 1.631, *p* = 0.014) were statistically different, showing that the SAMI significantly reduced SOL and improved SE in college students at week 3 under intervention. There were no statistical differences in other objective sleep indicators (TST, AT). In addition, there were differences in sleep quality among college students of different genders. Compared with females, males had worse SE (β = −3.245, *p* < 0.001), shorter TST (β = −14.582, *p* = 0.001), and more AT (β = 3.196, *p* = 0.001; Table 3).

**Table 3:** Linear mixed effect module analysis results-objective sleep quality.

	SOL		SE		TST		AT	
	*β*	*t* value	*β*	*t* value	*β*	*t* value	*β*	*t* value
Constant	9.896	11.453^***^	89.683	63.286^***^	337.693	40.713^***^	15.089	8.738^***^
*Time (week)*								
No-intervention	0.000	–	0.000	–	0.000	–	0.000	–
1	−1.274	−3.752^***^	0.368	0.554	6.106	1.939	0.741	1.505
2	−1.172	−3.451^**^	0.182	0.274	1.957	0.621	0.247	0.502
3	−1.658	−4.883^***^	1.631	2.455^*^	3.380	1.073	−0.173	−0.350
4	−1.624	−4.784^***^	0.623	0.938	2.722	0.864	0.138	0.280
*Gender*								
Male	0.857	1.864	−3.245	−4.365^***^	−14.318	−3.252^***^	3.196	3.426^**^
Female	0.000	–	0.000	–	0.000	–	0.000	–
*Grade*								
Freshman	−1.169	−1.281	0.901	0.610	13.789	1.578	−0.718	−0.388
Sophomore	−1.533	−1.778	1.031	0.740	−0.253	−0.031	−1.349	−0.771
Junior	0.000	–	0.000	–	0.000	–	0.000	–

^*^p<0.05; ^**^p<0.01; ^***^p<0.001.

### Effect of SAMI on mental status

#### Effect on anxiety

Compared with the no-intervention week, there were significant differences in T − AI in interventional weeks 1 to 4 (β = −1.023, *p* = 0.021; β = −1.086, *p* = 0.014; β = −1.343, *p* = 0.002; β = −2.418, *p* < 0.001, respectively), and S − AI in weeks 3 and 4(β = −1.933, *p* < 0.001; β = −2.730, *p* < 0.001). SAMI significantly decreased the T − AI of college students, and significantly decreased the S − AI in the 3rd and 4th weeks under intervention.

#### Effect on depression

Compared to the no-intervention week, there were significant differences in depression scores at weeks 1 to 4 (β = −1.774, *p* < 0.001; β = −1.009, *p* = 0.023; = −1.899, *p* < 0.001; β = −2.888, *p* < 0.001, respectively) under SAMI, showing that the SAMI significantly reduced college students’ depression scores. The results also show that boys had lower state anxiety (β = −4.153, *p* = 0.019), lower trait anxiety (β = −4.251, *p* = 0.009), and lower depression scores (β = −3.586, *p* = 0.021) than girls. Sophomores had higher state anxiety (β = 7.951, *p* = 0.017) and trait anxiety (β = 7.449, *p* = 0.015) than junior students (Table 4).

**Table 4:** Linear mixed effect module analysis results-mental status.

	S-AI		T-AI		BDI-II	
	*β*	*t* value	*β*	*t* value	*β*	*t* value
Constant	40.202	12.527^***^	41.361	14.044^***^	8.553	3.033^**^
*Time (week)*						
No-intervention	0.000	–	0.000	–	0.000	–
1	−0.773	−1.511	−1.023	−2.318^*^	−1.774	−4.021^***^
2	−0.862	−1.685	−1.086	−2.463^*^	−1.009	−2.286^*^
3	−1.933	−3.782^***^	−1.343	−3.044^**^	−1.899	−4.303^***^
4	−2.730	−5.339^***^	−2.418	−5.482^***^	−2.888	−6.545^***^
*Gender*						
Male	−4.153	−2.368^*^	−4.251	−2.639^**^	−3.586	−2.326^*^
Female	0.000	–	0.000	–	0.000	–
*Grade*						
Freshman	3.452	0.991	3.877	1.212	2.830	0.924
Sophomore	7.951	2.418^*^	7.449	2.467^*^	4.482	1.551
Junior	0.000	–	0.000	–	0.000	–

S-AI, State-Anxiety Inventory, T-AI; Beck Depression Inventory, BDI-II. ^*^p < 0.05; ^**^p < 0.01; ^***^p < 0.001.

#### Correlation between sleep quality and mental status

In order to investigate the correlation between the improvement of mental status and sleep quality, we conducted correlation analysis of the indicators that had significant changes after the intervention, and found that the subjective sleep quality PSQI was positively correlated with S-AI (r = 0.474), T-AI (r = 0.456), and BDI-II (r = 0.478), indicating that the higher the PSQI score, the higher the level of anxiety and depression; Sub-SOL was positively correlated with S-AI (r = 0.219), T AI (r = 0.200), and BDI-II (r = 0.144) too, indicating that the longer the time of Sub-SOL, the higher the level of anxiety and depression; In contrast, Sub-SE and Sub-TST were negatively correlated with S-AI (r = −0.236; r = −0.198), T-AI (r = −0.212; r = −0.166), and BDI-II(r = −0.153; r = −0.107), showing that the higher the SE and the longer the time of TST, the lower the level of anxiety and depression (Table 5).

**Table 5:** Analysis of the correlation between sleep quality and mental status.

	PSQI	Ob-SOL	Ob-SE	Sub-SOL	Sub-SE	Sub-TST	Sub-AT
S-AI	0.474^***^	−0.035	−0.047	0.219^***^	−0.236^***^	−0.198^***^	0.076
T-AI	0.456^***^	−0.034	−0.042	0.200^***^	−0.212^***^	−0.166^***^	0.059
BDI-II	0.478^***^	−0.008	0.005	0.144^***^	−0.153^***^	−0.107^**^	0.096^*^

Ob, Objective; Sub, Subjective ^*^p < 0.05; ^**^p<0.01; ^***^p<0.001.

### Minimum effective intervention length

**Figure 2:**
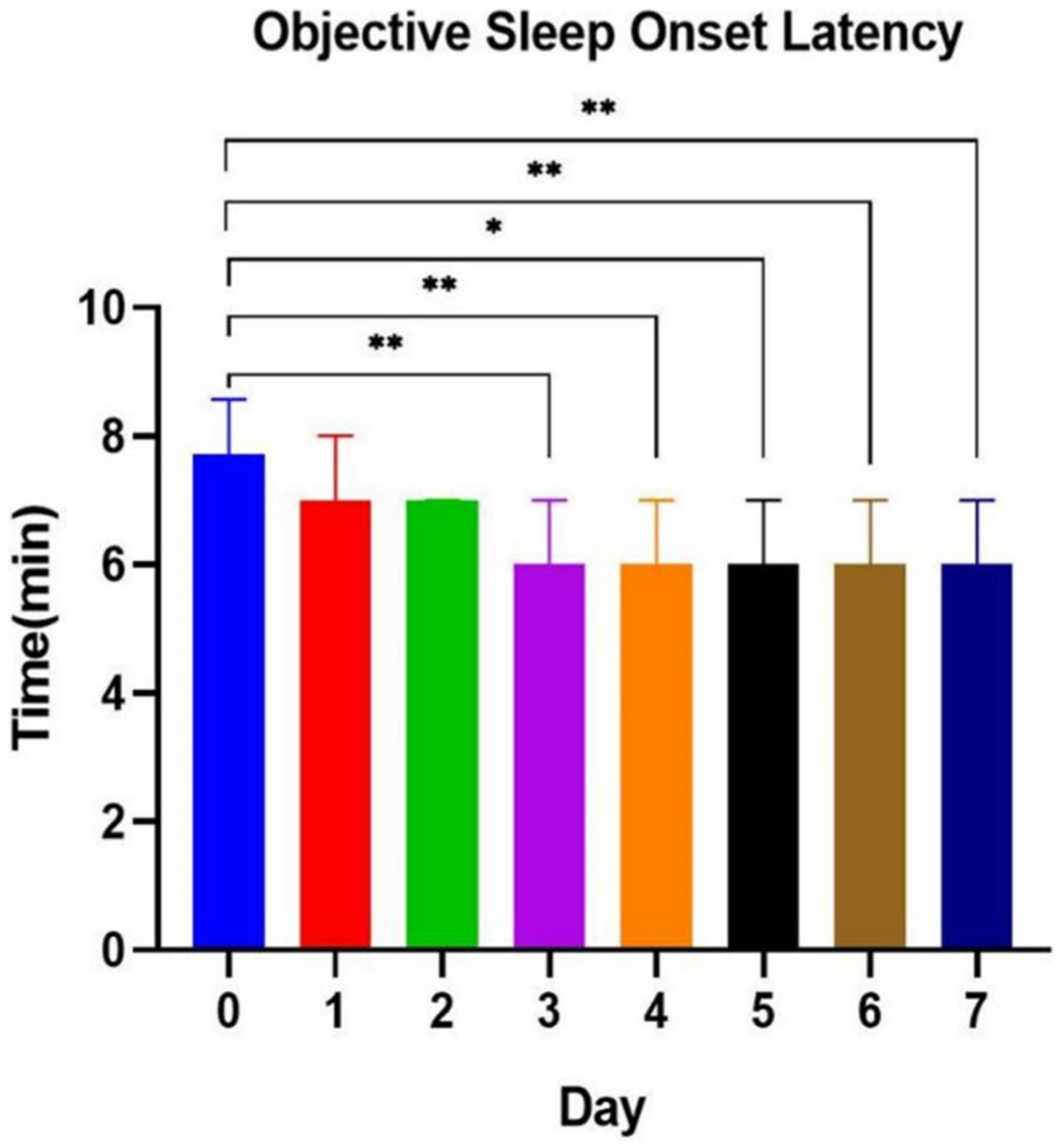
Objective sleep onset latency at music interventional day 1 to day 7. *p < 0.05; **p < 0.01; ***p < 0.001.

**Figure 3:**
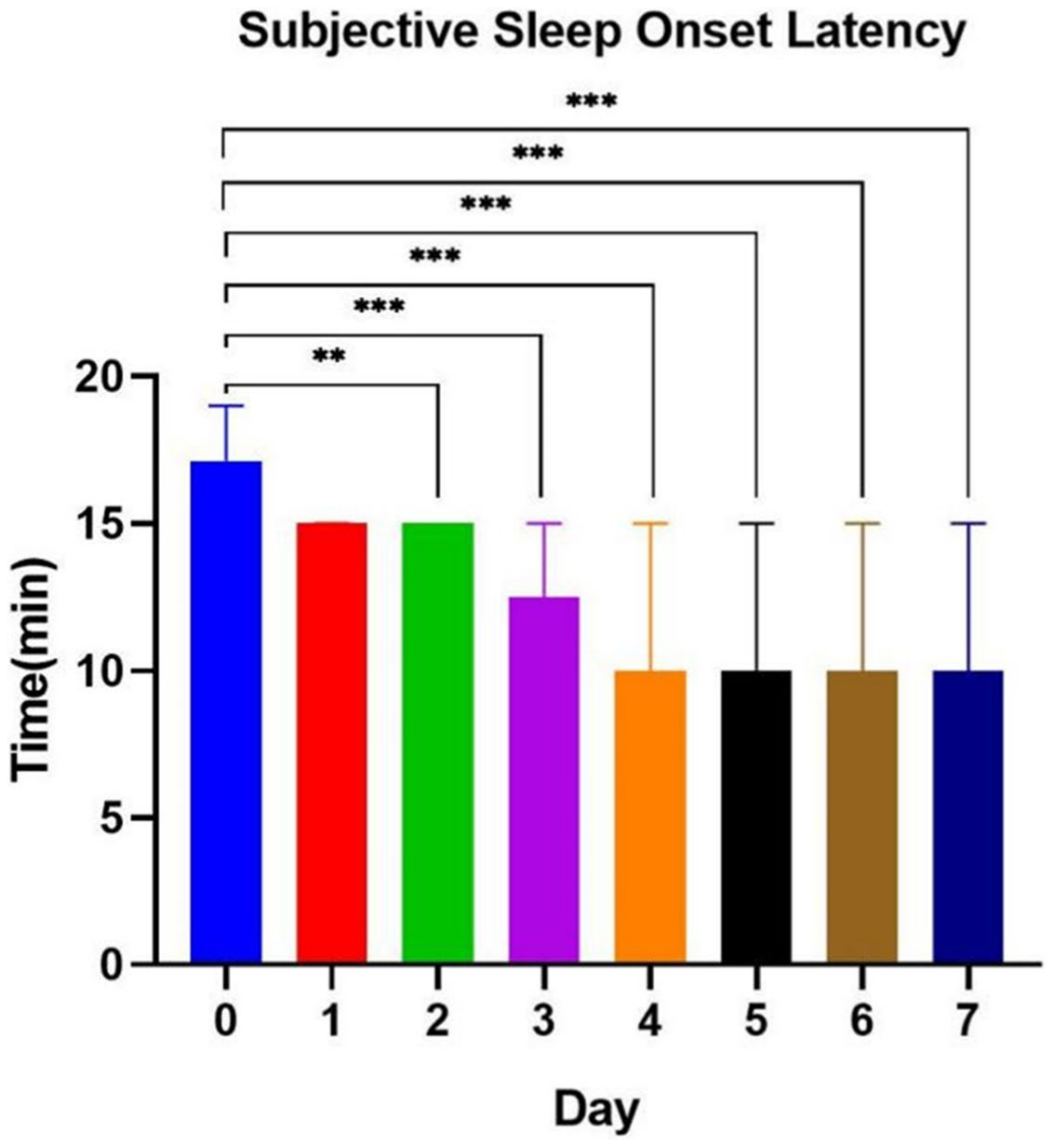
Subjective sleep onset latency at music interventional day 1 to day 7. *p<0.05; **p < 0.01; ***p < 0.001.

Based on the results of the mixed-effects model, subjective sleep quality PSQI scores and objective SOL were significantly different. In weeks 1 to 4 under SAMI, we conducted a daily analysis of subjective and objective SOL at interventional week 1. The results showed significant differences in objective SOL from days 3 to 7 under intervention compared to pre-intervention length (day 0; Figure 2), and in subjective SOL from interventional days 2 to 7 (Figure 3). Three days was the minimum effective intervention length to shorten the objective SOL, and 2 days was the minimum effective intervention length to shorten the subjective SOL.

## Discussion

The purpose of this exploratory study was to explore the effects of SAMI on the sleep quality and mental health of college students and to explore the minimum effective intervention duration. We found that sleep ambient music had a significant effect on enhancing subjective sleep quality, shortening objective SOL, and reducing anxiety and depression in college students. The outstanding effect of SAMI on objective SOL appeared on the 3rd day under SAMI, and it seemed to continuously improve the subjective and objective SOL after that. SAMI for more than 3 days is expected to become an effective treatment for shortening the SOL of college students. The results of our study are very important for lessening the difficulty in falling asleep and reducing the level of anxiety and depression of college students.

### Improvement of sleep quality

Previous studies found a reduction in subjective sleep quality PSQI scores (Yamasato et al., 2020) as well as in each dimension, an improvement in sleep quality (Harmat et al., 2008), and an increase in sleep satisfaction (Yamasato et al., 2020) among college students after music intervention, consistent with the results of our study. Previous studies showed that the main sleep problems of college students were difficulty in falling asleep (Reis et al., 2021), poor sleep quality (Chowdhury et al., 2020), and sleep pattern disorders (Li T. et al., 2020). In this study, we also found that difficulty in falling asleep is the most common sleep problem among college students (45.7%). SOL is an important indicator of the degree of difficulty in falling asleep. However, previous studies did not find that music intervention can significantly shorten objective SOL. The results of this study show that sleep ambient music is effective in shortening the subjective and objective SOL of college students. This finding may be unique. Elements of nature or environmental sounds contained in this type of music can activate memories and emotions (Braganca et al., 2015) and shift one’s attention away from stressful events to focus on the feelings brought by the music (Gao et al., 2009). This may be one of the principles by which environmental music improves sleep quality. Simultaneously, soothing melodies can also improve sleep quality by activating the parasympathetic nervous system and reducing sympathetic nervous system activity, regulating the balance between them to achieve physical and mental relaxation (Su et al., 2013). Trahan et al. (2018) study concluded that music chosen by the subjects themselves had more analgesic and anxiolytic effects compared to unfamiliar music. A review by Jespersen et al. (2022) suggested that music preference is one of the important factors influencing the effectiveness of interventions. Our sleep ambient music was chosen by the subjects themselves, thereby satisfying the subjects’ preferences. This may be another important factor that distinguishes the results of this study from previous studies. This study also showed that objective SE showed significant improvement only at week 3 under SAMI, but there was no significant change in objective TST or AT. This may be related to the management model of the college. However, in the subjective evaluation SE, TST, and AT all improved from the first week under SAMI and were maintained until the end of the intervention. Subjects included in the experiment were college students with sleep problems, weekly follow-up showed that most of them had high expectations for the experiment, and they expected the music to improve their sleep quality, that might lead to this difference from the objective results.

In addition, we found that freshmen and sophomores are at higher risk for poor sleep quality compared to seniors both in pre-and post-intervention, which may be related to their resilience to school and dependence on family (Li Y. et al., 2020; Sundas et al., 2020). The results also showed that boys had less SE, had shorter TST, and more AT compared to girls. However, the results of Becker et al. (2018), which showed that women had worse sleep quality, differed from ours. More researches are needed to demonstrate the effects of grade and gender differences on sleep quality of college students.

### Improvement of mental health

Sleep ambient music can partially reduce the degree of state anxiety, trait anxiety, and depression of college students. Chan’s study (Chan et al., 2010) among depressed patients found that listening to music for 20 min a day for two weeks significantly reduced depressive symptoms. Harmat et al. (2008) found that music decreased the level of depression in college students, a finding that was verified in this study. Possible reasons for music to reduce the level of depression include neurochemical changes and neuroplasticity. Music has been shown to modulate the release of neurotransmitters and hormones (e.g., dopamine, serotonin, and cortisol) thereby affecting depression (Sarkamo et al., 2013). And it can also stimulate neuroplastic changes in the brain, especially in areas involved in emotion processing and regulation (Koelsch, 2014).

In terms of anxiety improvement, a study by Burrai et al. (2020) found that music reduced the anxiety levels of subjects. In this study, further analysis revealed that trait anxiety lessened significantly during all 4 weeks under the music intervention, but abatement in state anxiety occurred only at week 3 and was maintained until week 4. The possible reason is that trait anxiety refers to the subject’s anxiety about their normal feelings, and as the subject’s sleep quality improves, trait anxiety lessens in parallel. However, state anxiety is different. In the early phase of study, factors such as the new sleep environment, musical intervention, and experimental administration may have caused relevant mental reactions in some subjects, triggering transient state anxiety (Kusumoto et al., 2022). As the experiment progressed, most subjects gradually adapted to the experimental procedure at week 3. This reinforced the effect of the music intervention so that abatement in state anxiety occurred at week 3 and was maintained until week 4. The different effects of SAMI on state anxiety and trait anxiety, and the relationship between the two under SAMI conditions, need further investigation.

Moreover, the results of this study show that sophomores have higher state anxiety and trait anxiety compared to freshmen and juniors from pre-intervention to post-intervention. This could be the result of selection bias resulting from the high percentage of sophomores in this study (62.3%). We also found that boys had lower levels of anxiety compared to girls. A cross-sectional survey of undergraduate students by Iqbal et al. (2015) found that girls scored higher than boys on anxiety, depression, and stress scores, similar to our findings. Further research is necessary to expound the effects of grade and gender differences on mental status of college students.

In this study, we performed SAMI for the college students only in the 30 min before bedtime. The effect on the modification of mental status was shown to be associated with the improvement in sleep quality. Okun et al. (2018) and McEvoy et al. (2019) studies found that poor sleep quality was associated with high anxiety-depression, consistent with our findings, which explained why the improvement in state anxiety in this study appeared after the improvement in sleep quality.

### Selection of intervention time

Chang’s study found that a 4-day musical intervention shortened the length of the non-rapid eye movement sleep-II and prolonged the duration of the rapid eye movement sleep period in subjects. Harmat et al. (2008) administered a 3-week music intervention to 35 college students and found that subjective sleep quality began to be improved at week 2 and was maintained until week 3, with significant improvement in depressive symptoms after the music intervention. Dickson et al. concluded that continuous music listening has a sustained improvement in sleep quality, and interventions of longer than 3 weeks may achieve long-term healthy sleep (Dickson and Schubert, 2020). A study by Chen et al. (2021) found that more than 4 weeks’ intervention was better than shorter interventions, and 4 weeks may be the optimal length of intervention for older adults. In this study, we explored the minimum effective duration of SAMI to improve sleep quality in college students, with the intention of providing a reference for follow-up studies and clinical applications. Based on the results, we concluded that the minimum effective intervention length for improving subjective SOL was 2 days and for improving objective SOL was 3 days. Therefore, we recommend the use of SAMI for more than 3 days to solve the sleep problems of college students who mainly have difficulty in falling asleep. It is noteworthy that the PSQI and SOL improved significantly from week 1 compared to pre-intervention, and the effect was maintained until week 4, while state anxiety showed significant improvement at week 3 and was maintained until week 4. Therefore, we suggest that continuous SAMI for more than 3 weeks may be more beneficial for the joint improvement of sleep quality and mental status. In addition, the cumulative and potential effects of music interventions are likely to be more sustained and extensive than existing results. Studies that have longer terms and more evaluation dimensions will be highly relevant in the future.

### Limitations and future research

Although we were fortunate to obtain some meaningful results, our study still suffers from the following shortcomings that need to be addressed head-on. First, in terms of experimental design, this study lacked a rigorous control group. We just compared the effects of different time points in the cycle SAMI on college students’ sleep quality and mental status. Second, in terms of sample selection, the subjects of this study were college students in medical school, which had certain specificities. Future studies should include more types of colleges and universities and more majors to facilitate a comprehensive understanding of the effects of SAMI. Finally, in terms of tools, the actigraphy was chosen to collect objective indicators of sleep in this study to truly reflect the effect of SAMI on the sleep quality of college students in real-life settings. The actigraphy has been recommended by the American Association of Sleep Medicine as an adjunct to sleep evaluation and is an effective means of assessing objective sleep parameters (Morgenthaler et al., 2007). It has various advantages, such as being easy to wear, not interfering with sleep, and allowing prolonged monitoring (Littner et al., 2003; Withrow et al., 2019). A retrospective study that included 27 outpatients with chronic insomnia comparing actigraphy with PSG found a good agreement coefficient (Martoni et al., 2012). However, the actigraphy still have shortcomings such as overestimation of sleep time and underestimation of wakefulness time.

Sleep ambient music used in this study can effectively shorten the SOL, improve the quality of sleep, and alleviate the level of anxiety and depressive symptoms of college students, and these effects are cumulative. We were the first to suggest that the minimum effective intervention length for SAMI to shorten SOL is 3 days, and to recommend SAMI for more than 3 weeks as a treatment for sleep problems combined with mental problems such as anxiety and depression. More randomized controlled trial (RCT)s are needed in the future, we will adopt a RCT to compare the effects of sleep ambient music with other music to obtain a more comprehensive evaluation and higher quality in terms of evidence-based medicine of the effects of SAMI on college students’ sleep. In addition, more multidimensional evaluations, longer-term interventions, and post-intervention follow-up will help to explore the long-term effects and other potential roles of SAMI. In the meantime, the effect of additional factors (e.g., gender) on sleep still deserves to be explored through better experimental tools and designs to explore its mechanisms. In the next study, we will add PSG in order to obtain more objective evidence. Especially in psychology, we were glad to find that sleep ambient music had a certain effect on the improvement of anxiety and depression in college students. Whether this effect is independent of the improvement of sleep quality, and whether sleep ambient music has a wider application prospect in promoting mental health, deserves further study.

## Data availability statement

The raw data supporting the conclusions of this article will be made available by the authors, without undue reservation.

## Ethics statement

The studies involving human participants were reviewed and approved by the Medical Ethics Committee of Army Medical University. The patients/participants provided their written informed consent to participate in this study.

## Author contributions

J-YL: conceived and designed the study. J-YL and S-PH: Music choice. W-HC, S-SL, TN, Y-ZX, and S-PH: experiments. W-HC and S-PH: data analysis. S-PH: drew figures and created tables and drafted the manuscript. J-YL and Y-MY: Reviewed and made improvements in the manuscript. All authors read and approved the final manuscript.

## Funding

This work was supported by Army Medical University of Humanities and Social Sciences Foundation of China (Grant number 2021XRW01).

## Conflict of interest

The authors declare that the research was conducted in the absence of any commercial or financial relationships that could be construed as a potential conflict of interest.

## Publisher’s note

All claims expressed in this article are solely those of the authors and do not necessarily represent those of their affiliated organizations, or those of the publisher, the editors and the reviewers. Any product that may be evaluated in this article, or claim that may be made by its manufacturer, is not guaranteed or endorsed by the publisher.
