# Supplementary material for: Effect of sleep ambient music on sleep quality and mental health in college students: a self-controlled study
Source: Front Psychol. 2023 Jul 7;14:1171939. doi: 10.3389/fpsyg.2023.1171939 (PMC10361298; doi:10.3389/fpsyg.2023.1171939)
Supplement: Supplementary file 1 [file Table_1.DOCX]

TABLE 1 Characteristics of respondents.

| **Characteristics(n=138)** | |
| --- | --- |
| **Age; mean (SD)** | 20.029（1.255） |
| **Gender** |  |
| Male | 73（52.9%） |
| Female | 65（47.1%） |
| **Grade** |  |
| Freshman | 41（29.7%） |
| Sophomore | 86（62.3%） |
| Junior | 11（8.0%） |
| **Sleep problems (Multiple choice)** | |
| Difficulty falling asleep | 69（45.7%） |
| Difficult to maintain sleep | 32（21.2%） |
| Easy awakening | 29（19.2%） |
| Other | 11（7.3%） |
| Nothing | 10（6.6%） |
| **Factors affecting sleep (Multiple choice)** | |
| Pressure of life or study | 97（32.6%） |
| Psychological factors | 78（26.2%） |
| Irregular life | 50（16.8%） |
| Environmental impact | 47（15.8%） |
| Nervous fragility | 21（7.0%） |
| Other | 5（1.7%） |
| **Measures to deal with sleep disturbance (Multiple choice)** | |
| Listen to the music | 71（31.1%） |
| play with the mobile phone | 64（28.1%） |
| Watching TV or books | 23（10.1%） |
| Counting sheep | 18（7.9%） |
| Use of drugs | 10（4.4%） |
| Other | 10（1.4%） |
| Nothing | 32（14.0%） |
| **PSQI; mean (SD)** | 8.928（2.866） |
| **Emotion related indicators; mean (SD)** | |
| S-AI | 44.333（10.456） |
| T-AI | 46.022（9.500） |
| BDI | 11.159（9.355） |
| Note: *SD* refers to standard deviation. | |

The multiple choice questions in the general data questionnaire (sleep problems, factors affecting sleep, methods taken to deal with sleep problems) and whether the proportion distribution of each option was uniform or not were analyzed by the chi-square goodness-of-fit test.
